# Libertellenone H, a Natural Pimarane Diterpenoid, Inhibits Thioredoxin System and Induces ROS-Mediated Apoptosis in Human Pancreatic Cancer Cells

**DOI:** 10.3390/molecules26020315

**Published:** 2021-01-09

**Authors:** Weirui Zhang, Yuping Zhu, Haobing Yu, Xiaoyu Liu, Binghua Jiao, Xiaoling Lu

**Affiliations:** 1 College of Basic Medical Sciences, Department of Biochemistry and Molecular Biology, Naval Medical University, Shanghai 200433, China; weiruizh@163.com (W.Z.); yuhaobing1986@126.com (H.Y.); biolxy@163.com (X.L.); 2 College of Basic Medical Sciences, Experimental Teacher Center, Naval Medical University, Shanghai 200433, China; zhuyuping72@hotmail.com

**Keywords:** Libertellenone H, pancreatic cancer, ROS, Trx system, apoptosis

## Abstract

Libertellenone H (LH), a marine-derived pimarane diterpenoid isolated from arctic fungus *Eutypella* sp. D-1, has shown effective cytotoxicity on a range of cancer cells. The present study is to explore the anticancer effect of LH on human pancreatic cancer cells and to investigate the intracellular molecular target and underlying mechanism. As shown, LH exhibited anticancer activity in human pancreatic cancer cells by promoting cell apoptosis. Mechanistic studies suggested that LH-induced reactive oxygen species (ROS) accumulation was responsible for apoptosis as antioxidant N-acetylcysteine (NAC) and antioxidant enzyme superoxide dismutase (SOD) antagonized the inhibitory effect of LH. Zymologic testing demonstrated that LH inhibited Trx system but had little effect on the glutathione reductase and glutaredoxin. Mass spectrometry (MS) analysis revealed that the mechanism of action was based on the direct conjugation of LH to the Cys^32^/Cys^35^ residue of Trx1 and Sec^498^ of TrxR, leading to a decrease in the cellular level of glutathione (GSH) and activation of downstream ASK1/JNK signaling pathway. Taken together, our findings revealed LH was a marine derived inhibitor of Trx system and an anticancer candidate.

## 1. Introduction

The thioredoxin system, composed of thioredoxin (Trx), thioredoxin reductase (TrxR), and nicotinamide adenine dinucleotide phosphate (NADPH), which is a critical antioxidant system in defensing against oxidative stress and maintaining cellular redox homeostasis by eliminating redundant ROS [1,2]. In mammals, Trx1 and TrxR1 were the dedicated isoforms of Trx and TrxR for predominantly cytosolic localization. Structurally, Trx contains a highly conserved -Cys-Gly-Pro-Cys- sequence in their catalytic center, and TrxR has a conserved -Gly-Cys-Sec-Gly sequence in the *C*-terminal redox center [3]. The critical cysteine and selenocysteine motifs can modulate specific signal transduction cascades through its redox sensitive sulfhydryl switches [4]. The active reduced Trx (Trx-(SH)_2_) with vicinal dithiol can interact with downstream proteins via thiol–disulfide exchange reactions to form a disulfide bond and oxidized Trx (oxidized (Trx-S_2_)). The oxidized inactive disulfide of Trx-S_2_ is then recycled to reduced Trx-(SH)_2_ by TrxR at the expense of NADPH [1,5].

Accumulating evidence suggests that Trx system plays a key role in tumor initiation, progression and drug resistance. In the meantime, cancer cells are highly dependent on the Trx system. For example, transfection with Trx1 increases NIH 3T3 cell proliferation and stimulates tumor formation of MCF-7 breast cancer cells, whereas transfection with dominant-negative mutant Trx1 (C^32^S/C^35^S) delays tumor progression and metastasis [6]. Moreover, TrxR deficiency reversed the phenotype [7] and tumorigenicity of malignant cells rather than simply reducing tumor progression and metastasis in murine tumor cells [8]. The reduced Trx1can bind to the apoptosis signal regulating kinase (ASK1) directly and tumor suppressor PTEN to help tumor cells evade apoptosis [9,10]. ASK1, a member of mitogen activated protein kinase (MAPK) kinase kinases family, activates the c-Jun *N*-terminal kinases (JNK) and p38 MAPK pathway which is the major component for tumor necrosis factor -α mediated apoptosis in response to oxidative stress [11]. Further, the elevated levels of Trx system stimulates angiogenesis by the induction of hypoxia-inducible factor 1 [12,13] and vascular endothelial growth factor, and the Trx-dependent heme oxygenase-1 pathway [14]. It has been reported that overexpression of Trx system was found in many human cancer cell lines and human tumors, like lung, breast, colorectal, hepatocellular, pancreatic and gastric carcinoma [15,16,17,18]. In addition, the high expression levels of Trx system in cancer cells are linked to aggressive tumor growth and clinically correlated to poor prognosis. Hence, targeting the Trx system is a promising strategy for cancer treatment [5,19].

Up to now, several small molecules targeting either Trx1 or TrxR have been developed as potential therapy for cancer [5,19,20]. Because thiol groups especially the selenothiols are essential groups for their activity, most inhibitors selectively target *C*-terminal (GC^497^U^498^G) of TrxR and redox active site (C^32^GPC^35^) of Trx1 [21]. There are many inhibitors of TrxR that based on natural products such as curcumin [22], shikonin [23], myricetin, and quercetin [24]. It’s worth noting that brevetoxin-2, a neurotoxin produced by the Florida red tide organism *Karenia brevis*, was the only marine-derived TrxR inhibitor exhibiting a specific inhibitory mechanism [25]. Compared to TrxR, less natural inhibitors have been developed to target Trx1. Isoforretin A, a diterpeniod from the leave of *Isodon forrestii* var [26]. *forrestii*, specially inhibited Trx1 and mediated anticancer effects in preclinical settings. Then, diallyl trisulfide (DATS), known as one of the main ingredients of galic, suppressed interaction of Trx1 and downstream nuclear factor kappa B. Of note, a small molecular PX12 that covalently bind to non-catalytic Cys^73^ of Trx1 has failed in phase II trials for pancreatic cancer due to lack of significant anti-tumor activity [27]. Currently, there is no clinically available anticancer drug that specifically targets the Trx system and no marine natural products have shown specific inhibitory effect on Trx1. Therefore, it is worthy to search novel marine natural Trx1 and TrxR inhibitors for cancer therapy.

The particularity of the marine ecological environment (high pressure, high salt, and hypoxia) makes the secondary metabolites of marine microorganisms have unique structures with extensive biological activities, which may have profound effects for drug discovery and development [28]. In recent years, more than 1000 marine natural products have been newly discovered every year, and about half of these were obtained from marine microorganisms [29]. To date, the marine microorganism-based drugs rifampicin, cephalosporin, and marizomib are clinical available, and plinabulin has completed its phase III clinical study, highlighting the potential of marine microorganism natural compounds [30].

In our previous studies, a series of new pimarane diterpenes were isolated from *Eutypella* sp. D-1, an arctic fungus from the soil of high latitude of Arctic. Among them, Libertellenone H showed more significant anti-proliferative effects on a broad spectrum of tumor cells than its homologs. However, the anticancer mechanism of LH has not been fully studied [31]. In this paper, we demonstrated LH induced ROS accumulation by suppressing Trx1 and TrxR through binding to the Cys^32^/Cys^35^ residues of Trx1 redox sites and alkylating the *C*-terminal redox-active site Sec^498^ of TrxR, resulting in ASK1/JNK signal activation and apoptosis in cancer cells.

## 2. Results

### 2.1. LH Inhibits Cell Growth in Human Pancreatic Cancer Cell Lines

**Figure 1:**
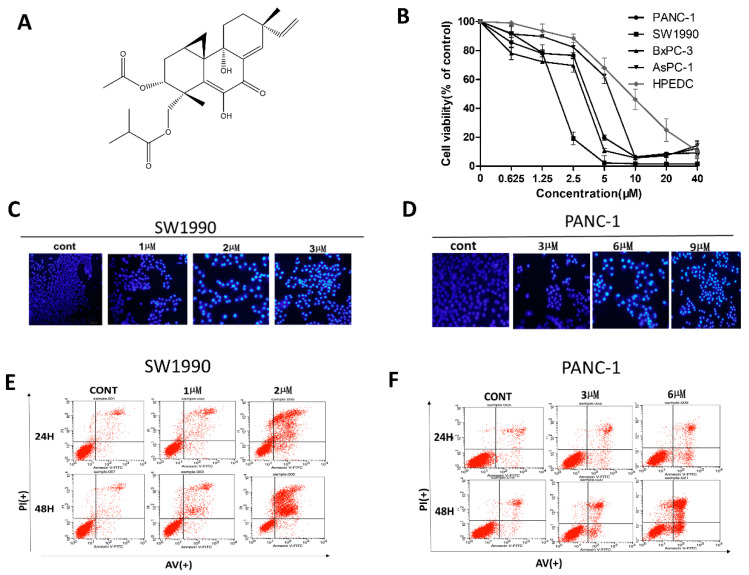
Effects of LH on human pancreatic cancer cell lines. (**A**), Chemical structure of LH. (**B**), Growth inhibition of LH on human pancreatic cancer cell lines PANC-1, SW1990, AsPC-1, BxPC-3 and human pancreatic duct epithelial cells HPDE6-C7. Cells were incubation with 0, 0.625, 1.25, 2.5, 5, 10, 20, 40 µM LH for 48 h and cell viability was detected by the CCK8 assay. (**C**,**D**), PANC-1 and SW1990 cells were exposed to indicated concentrations of LH for 48 h, and morphological changes were indicated by Hoechst 33258 staining analysis. (**E**), SW1990 cells were exposed to 0, 1, 2 µM LH for 24 h or 48 h. Apoptosis was analyzed by flow cytometry after Annexin V-FITC/PI staining. (**F**), PANC-1 cells were treated with 0, 3, 6 µM LH for 24 h or 48 h. Apoptosis was analyzed by flow cytometry after Annexin V-FITC/PI staining.

In our previous studies, LH had been identified as an effective anti-tumor agent with IC_50_ value in the low micromolar concentration range in various cancer cell lines, including MCF7, U251, SG7901, HUH-7, HeLa, and H460 [31]. Here, we further investigated the inhibitory effects of LH on several human pancreatic cancer cells, a malignant tumor with a very low five-year survival rate. We evaluated the cytotoxicity of LH in four pancreatic cancer cell lines, PANC-1, SW1990, AsPC-1, and BxPC-3 and human pancreatic duct epithelial cells HPDE6-C7 via the CCK8 assay. As exhibited in Figure 1B, LH displayed a preferential anti-proliferative activity against the four pancreatic cancer cell lines in a dose-dependent manner with IC_50_ values of 3.21, 0.67, 2.78, and 5.53 µM in PANC-1, SW1990, AsPC-1, and BxPC-3 respectively after 48 h. The IC_50_ value of LH for HPDE6-C7 was 10.86 µM.

Next, we examined the effect of LH on the induction of apoptosis in PANC-1 and SW1990. Hoechst staining results in Figure 1C,D showed that apoptosis cells with chromatin condensation and fragmentation markedly increasing in both PANC-1and SW1990 cells by LH after 48 h treatment. The Annexin V-FITC/PI double staining assay further made a quantitation evaluation of apoptosis. Under the condition of 2 µM LH for 48 h, the number of early and late apoptosis of SW1990 cells is 47.9%; under the condition of 6 µM LH for 48 h, the ratio of early and late apoptosis PANC-1 cells is 44.5% (Figure 1E,F). It’s illustrated that, compared to the control group, LH triggered the apoptosis of PANC-1 and SW1990 in a time and concentration dependent manner.

### 2.2. LH Induced-ROS Accumulation Involved in Its Antitumor Activity

**Figure 2:**
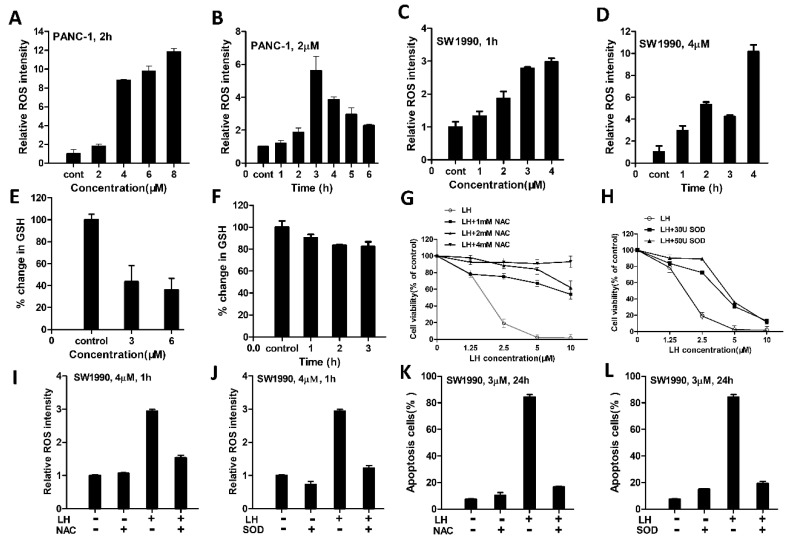
LH induced-ROS accumulation is involved in its antitumor activity. (**A**), ROS levels in PANC-1 cells were assessed after 0, 2, 4, 6, 8 µM LH treated for 2 h by fluorescent probe DCFH/DA staining and determined by flow cytometry. (**B**), PANC-1 cells were incubation with 2 µM LH for 0, 1, 2, 3, 4, 5, 6 h, and ROS levels were measured as mentioned above. (**C**), SW1990 cells were treated with 0, 1, 2, 3, 4 µM of LH for 1 h and then ROS levels were assessed as mentioned above. (**D**), SW1990 cells were treated with 4 µM LH for 0, 1, 2, 3, 4 h, and then ROS levels were measured as mentioned above. (**E**), LH dose dependently decreased intracellular GSH levels. GSH levels were measured after SW1990 cells treated with 0, 3, 6 µM LH for 2 h by GSH and GSSG Assay Kit. (**F**), LH decreased intracellular GSH levels. GSH levels were determined after SW1990 cells disposed to LH at 4 µM for 0, 1, 2, 3 h. G-L, SW1990 cells were pretreated with NAC, SOD at indicated concentration for 30 min, the cell viability (**G**,**H**) was determined by CCK8 assay after incubated with 0, 1.25, 2.5, 5, 10 µM LH for 48 h, the levels of ROS (**I**,**J**) were determined by flow cytometry after incubated with 4 µM LH for 1 h, the apoptosis (**K**,**L**) was assessed by flow cytometry after incubated with 3 µM LH for 24 h.

It has been reported induction of ROS preferably induced cancer cell apoptosis [32]. Therefore, the intracellular ROS level was determined using the fluorescent probe dichloro-dihydro-fluorescein diacetate (DCFH/DA) to detect induction of ROS after exposure to LH in SW1990 and PANC-1 cells. As shown in Figure 2A,B, compare to the control groups, the intracellular ROS level in PANC-1 and SW1990 cells are both increased after treated with LH (Figure 2C,D).

As an important antioxidant in cells, GSH plays a critical role to prevent oxidant damage and adjust redox homeostasis. Then, the effect of LH on intracellular GSH level was examined. Figure 2E,F showed that compared with the control group, intracellular GSH level decreased after LH treatment in SW1990 cells. Next, to determine increased intracellular ROS levels may act on LH-induced cell growth inhibition or apoptosis, the antioxidant NAC and antioxidant enzyme SOD was added to the cancer cell lines before further LH treatment. The results showed that pretreatment with NAC or SOD partly counteracted LH-induced cell growth inhibition (Figure 2G,H), intracellular generation of ROS (Figure 2I,J), and apoptosis in SW1990 cells. (Figure 2K,L). These data suggested that the accumulation of ROS might be responsible for LH-induced cancer cells growth suppression.

### 2.3. LH Inhibited Trx/TrxR System

LH contained two α, β-unsaturated carbonyl groups, which can interact with free thiol of cysteine and selenocysteine residues in proteins by forming covalent linkages or offering an electrophilic center in oxidation-reduction reactions.

**Figure 3:**
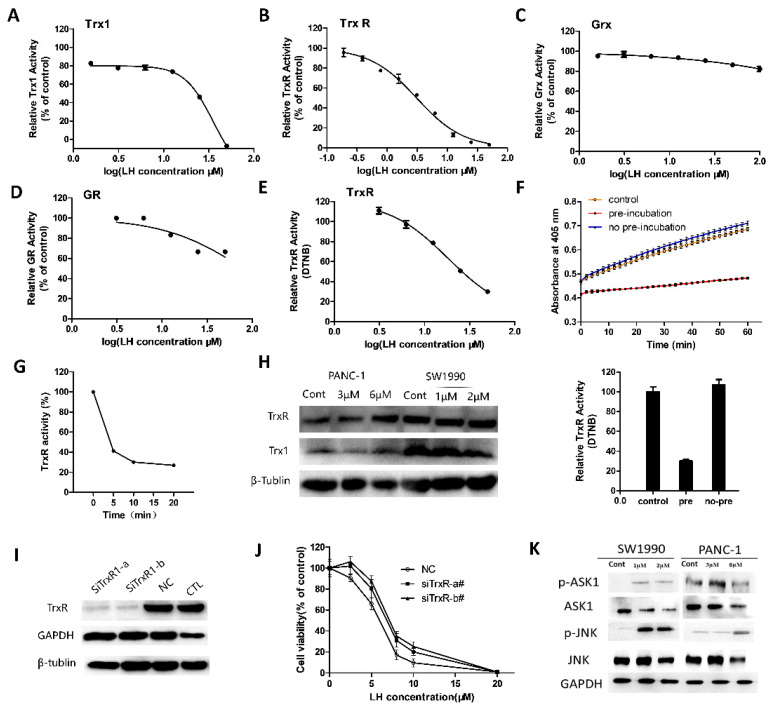
LH exhibited effective inhibition on Trx/ TrxR system. In vitro assays for Trx1 (**A**), TrxR (**B**), Grx1 (**C**), GR (**D**). For Trx1 activity assay, 0.04 µM Trx1, 1 µM TrxR was incubated with LH. For TrxR activity assay, 0.4 nM of TrxR, 2 µM TrxR was incubated with LH. For Grx activity assay, 0.6 nM of hGrx-1, 50 nM GR, 0.5 mM GSH was incubated with LH. For GR activity assay, 20 µM of GR, 1 mM GSH was incubated with LH. (**E**), TrxR was pre-reduced with NADPH for 30 min with indicated concentrations of LH, followed by addition of DTNB. (**F**), 50 µM LH was pre-incubated or no pre-incubated with TrxR for 30 min, followed by addition of DTNB. (**G**), NADPH-reduced TrxR was incubated with 10 µM LH for 0, 5, 10, 20 min. (**H**), Expression levels of Trx1 and TrxR1 in SW1990 and PANC-1 cells were determined after incubating with indicated concentration of LH for 24 h by Western blotting. β-tublin served as protein-loading control. (**I**), Knockdown efficiency was analyzed after SW1990 cells transfected with TrxR siRNA, control siRNA and positive GAPDH siRNA for 48 h by Western blotting. (**J**), TrxR-silenced SW1990 cells and control cells were treated with 0, 2.5, 5, 8, 10, 20 µM LH for 48 h, and cell viability was measured by CCK8 assay. (**K**), SW1990 and PANC-1 cells were incubated with indicated concentration LH for 48 h. Total and activation forms of ASK1 and JNK were evaluated by Western blotting. GAPDH is shown as a protein-loading control.

So, we examined whether LH was a novel inhibitor of Trx1 and TrxR. First, in cell free system we detected the effects of LH on Trx1/TrxR using commercially available kits which is based on fluorescent substrate reduced by cooperation of Trx1 and TrxR. The investigation of inhibition potency of LH toward Trx1 is measured by reaction 1, and TrxR is measured by reaction 2. The results suggested LH significantly inhibited Trx1 and TrxR enzymes with IC_50_ value of 35.15 µM and 3.10 µM in Trx system (Figure 3A,B). To investigate the specificity of LH on Trx system, the effects of LH on some other thiol-containing enzymes, human glutaredoxin 1 (Grx1) and glutathione reductase (GR) were analyzed. The results showed that LH almost had no inhibitory effects on Grx1 (Figure 3C) and very weak inhibition on GR (Figure 3D).

(1) $$NADPH+H^{+}+Insulin-S_{2}\overset{Trx+TrxR}{\to }NADP^{+}+Insulin-{\left(SH\right)}_{2}$$

(2) $$NADPH+H^{+}+fGSSG\overset{TrxR+Trx}{\to }NADP^{+}+2fGSH$$

In addition to Trx, human TrxR has an extensive substrate specificity including induced disulfides of low molecular weight. A classical way to examine the activity of the TrxR is by using 5,5’-dithiobis-(2-nitrobenzoic acid (DTNB)) with NADPH as the ultimate electron donor. Therefore, we determined the activity of TrxR in reduction of low molecular weight disulfide of DTNB in the presence and absence of LH (Reaction 3). The analysis results in Figure 3E showed that LH also effectively inhibited TrxR in the reduction of DTNB with IC_50_ value of 18.26 µM. When no pre-incubation of TrxR and LH before the reaction, the reduction of DTNB did not occurred (Figure 3F). Then, after 5 min of incubation, TrxR activity was almost completely inhibited by LH (Figure 3G), indicating that the alkylation of TrxR by LH occurs correlated with incubation time and slowly relative to the reduction of DTNB. Those results of experiment showed that LH is an efficient inhibitor of Trx/ TrxR system.

(3) $$NADPH+H^{+}+DTNB\overset{TrxR}{\to }NADP^{+}+2TNB$$

Figure 3H showed that compare to the control group, the expression levels of Trx1 ware decreased in LH-treated PANC-1 and SW1990 cells, while there were no significant changes of TrxR in both cell lines. Next, to evaluate the role of Trx system in LH-induced cell growth inhibition and apoptosis, siRNA was used to knockdown TrxR expression in cells. The data in Figure 3H, I show that the protein expression level of TrxR decreased in the siRNA-TrxR interference SW1990 cells. As shown in Figure 3J, knockdown of TrxR antagonized the proliferation inhibition effect of LH, rendering siRNA-TrxR interference cells less sensitive to LH. It’s suggested that the expression levels of TrxR influenced cell sensitivity to LH.

Reduced Trx can bind to ASK1 and suppress its kinase activity to avoid triggering downstream signaling and ASK1-induced apoptosis. Once ASK1 is dissociated from Trx, subsequently ASK1/JNK signaling cascade is activated. Therefore, the effects of LH treatment on the ASK1-JNK signaling in PANC-1 and SW1990 cells were determined (Figure 3K). Indeed, the Western blot analysis results demonstrated the increased phosphorylation of ASK1 and JNK following by LH treatment, indicating that the phosphorylation and activation of ASK1/JNK signaling may be involved in LH-induced apoptosis in cancer cells.

### 2.4. LH Covalently Bound to Cys^32^ and Cys^35^ Residues of Trx1

**Figure 4:**
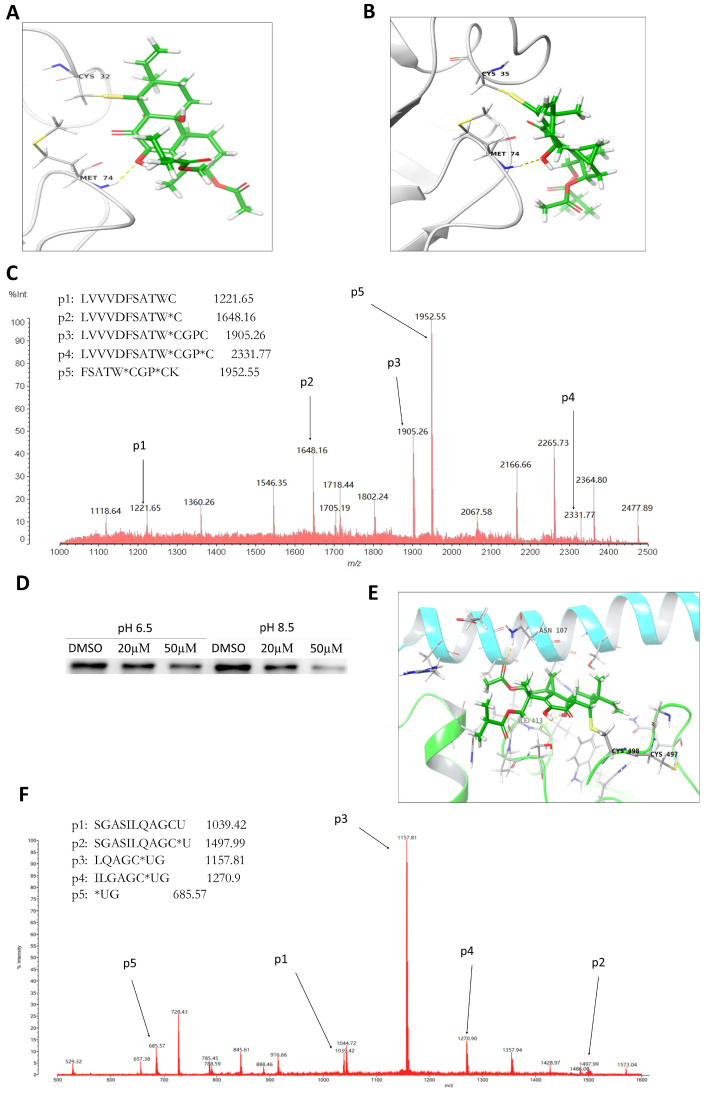
LH covalently binds toTrx1 and TrxR. Binding interactions between LH and Cys^32^ residues (**A**) or Cys^35^ residues (**B**) of Trx1 by molecular docking. (**C**), The adduct was detected by MS/MS after LH (5 mM) incubating with Trx1 (35 µg) at 37 °C for 2 h. (**D**), LH was incubated with NADPH and TrxR at 37 °C for 1 h, then added to BIAM alkylation at pH 8.5 or 6.5. The same amount of dimethyl sulfoxide (DMSO) was used as a control. (**E**), Binding interactions between LH and Cys^498^ residues of TrxR. (**F**), The adduct of LH and TrxR was detected by MS/MS after LH (3.3 mM) incubating with TrxR (4.8 µg) at 37 °C for 1 h.

In order to explore the interactive detail of LH and Trx1, molecular docking analysis were used to predict the binding modes between LH and Trx1. The energetically optimized binding mode of LH and Trx1 was shown in Figure 4A,B. The strong covalent bond between free sulfhydryl of reactive residues Cys^32^/Cys^35^ and α, β-unsaturated carbonyl group of LH was formed through Michael addition. Meanwhile, it can be found that the hydroxyl group of LH formed hydrogen bond with Met^74^ of Trx.

To confirm the directly combination between LH and Trx1 further, LH and *h*-Trx1 enzyme were incubated and the products were determined by LC-MS/MS (Figure 4C). The molecular weight of LH was 458.24. The component at m/z 1221.65 corresponding to the 22 to 32 residues in Trx1 as LVVVDFSATWC, compared to the component at *m/z* 1648.16 corresponding to the same peptide plus one reducible LH (the two hydroxyl groups reduced to hydrogen atoms) molecule, which indicated forming a covalent complex between LH and Cys^32^ of Trx1. In the same way, the component at *m/z* 1905.26 and 2331.77 represented the same peptide LVVVDFSATWCGPC and peptide plus one reducible LH, indicating LH covalently conjugated at Cys^35^. In general, the MS/MS analysis demonstrated that the covalent bonds formed between LH and Trx1 at the ratio of 2:1, and both Cys^32^ and Cys^35^ residues of Trx1 were targets for LH.

### 2.5. LH Covalently Bond to Sec^498^ Residues of TrxR

In the reduced form of TrxR, Cys^497^ and Sec^498^ in C-terminal redox active sites were present as -SH/-SeH group. The pKa value of Cys-SeH and most protein Cys-SH residues are about 8.5 and 5.7 respectively [33]. Thus the -SH/SeH groups in human TrxR can selectively be labeled with biotin-conjugated iodoacetamide (BIAM) at different pH buffers. At pH 8.5, both the -SH and -SeH groups are alkylated, and at pH 6.5, only the -SeH group is alkylated. As shown in Figure 4D, at pH 6.5 the labeling intensity were significantly weaker than control, demonstrating that Sec^498^ of TrxR can be alkylated by LH prior to BIAM. At pH 8.5, the weakened extent of labeling intensity was same as the extent observed in pH 6.5, indicating that the Sec^498^ of TrxR *C*-terminal redox-active site was specifically for LH modification.

To investigate the covalently binding models between LH and TrxR, molecular docking studies were performed. In our docking studies, the Sec^498^ residue was altered to Cys residue in the crystallographic structure of TrxR to accommodate calculation of software. Mainly combination modes were shown in Figure 4E, the covalent binding is more likely between Cys^498^ and α, β-unsaturated carbonyl group of LH through Michael addition reaction. Meanwhile, two hydrogen bonds formed between the ketonic oxygen of LH with Asn^107^ and hydroxyl at the carboatomic ring of LH with Ile^413^.

To further confirm LH binding to TrxR directly, LH incubated with TrxR protein, and tested by MS analysis (Figure 4F). TrxR protein was incubated with LH at 37 °C for 2 h before MS analysis. The molecular weight of LH was 458.24. The component at *m/z* 1039.42 corresponding to the *N*-terminal 487 to 498 residues of TrxR, represented the peptide SGASILQAGCU. The molecular weight of this peptide plus one molecular LH was equal to the molecular weight of component at *m/z* 1497.99. However, no signal of any peptide plus two LH molecules was detected, suggesting that an equivalent amount of LH covalent bound to the TrxR. The results suggested that Sec^498^ of TrxR was the specific target for TrxR.

## 3. Discussion

In the present study, we evaluated the antitumor potentials of LH, a pimarane diterpenoid from Arctic fungus *Eutypella* sp. D-1. It’s shown that LH, as a Trx system inhibitor, triggers potent ROS-mediated apoptosis in human pancreatic cancer cell lines.

The inhibitory effect of LH on Trx system in pancreatic tumor cell lines was demonstrated by the followings. First, the ROS level can be rapidly increased and maintained at a high level for a long time with the intracellular reduced GSH was depleted after LH treatment. Second, the ROS scavenger NAC and SOD reversed LH-elicited apoptosis, demonstrating that excessive ROS generation underlie the proapoptotic activity of LH. It’s suggested that LH regulated cellular antioxidant systems, and the collapse of redox homeostasis may be account for LH induced cell growth inhibition and apoptosis. Third, in the cell-free system LH inhibited Trx1 and TrxR enzymes with IC_50_ value of 35.15 μM and 3.10 μM, but had little effects on Grx and GR in the cell-free system. Besides, downregulation of TrxR expression in SW1990 cells decreased LH-induced growth-inhibitory effects. The downstream signaling pathway of Trx system, ASK1/JNK cascade, associated with apoptosis was activated after LH treatment. Importantly, LH-Trx1 adducts and LH-TrxR adducts were tested by MS/MS analysis. It’s revealed that the Cys^32^/ Cys^35^ residues at the active sites of Trx1 and Sec^498^ at *C*-terminal redox-active site of TrxR were identified as binding sites of LH, suggesting LH was a Trx and TrxR inhibitor.

In cancer cells, the metabolic abnormalities and oncogenic signaling are always accompanied by the excess ROS accumulation and trigger a redox adaptation response, resulting an increased antioxidant capacity, such as overexpressing of ROS scavengers including Trx and/or GSH and a shift of redox dynamics with exorbitant ROS generation and elimination to keep the ROS levels below the toxic threshold [32,34]. Therefore, cancer cells would be more dependent on the antioxidant system and more susceptible to induce further oxidative stress when exposed to exogenous ROS-generating agents or compounds that inhibit the antioxidant system [32,35]. In our work, the effect of LH on cancer cells included cell growth inhibition, apoptosis and excess generation of intracellular ROS. Those effects can significantly offset by antioxidants NAC and SOD. As such, ROS accumulation may be in the key position in mediating anticancer activity of LH.

Grx system, whose function is the same as Trx system through thiol-disulfide exchange, is consisted of Grx1, GSH, GR, and NADPH [1]. As LH showed stronger suppression effect against Trx/TrxR than Grx/GR, we propose that LH showed a relatively specialty on Trx/TrxR system as following. (1) GR and TrxR both belong to the pyridine nucleotide disulfide oxidoreductase family of dimeric flavoenzymes. TrxR possessed two active sites containing the catalytic group of disulfide or seleno-sulfide: *N*-terminal active center (-Cys^59^-Val-Asn-Val-Gly-Cys^64^) and the *C*-terminal active center (Cys^497^-Sec^498^-Gly-). Compare to the TrxR, GR has similar *N*-terminal active center domain and lacks the *C*-terminal active center [1,36]. According to the stronger suppression effect of LH on TrxR than GR, it’s deduced that LH targeted *C*-terminal active site containing the catalytic group of disulfide or seleno-sulfide of TrxR. Furthermore, BIAM-labeling assays proved the Sec^498^ of *C*-terminal domain as the primary site of alkylation by LH, which is verified by LC-MS/MS analysis and molecular docking simulation. Those results demonstrated that LH selectively targeted towards the Sec^498^, and showed fairly specificity on TrxR. (2) Trx is the parent of a family of oxidoreductases whose function carried on by thiol-disulfide exchange including Grx. Trx and Grx have a similar three-dimensional topology and active site CXXC motif. Some researches indicated that the reactivity of thiol-containing enzymes depends not only on their –CXXC residues, but on the spatially close to the redox sites [37,38]. The hydrogen bond and salt bridge forming in the active site play an important role in the function and activity of the enzyme. In our study, besides the Cys^32^/Cys^35^ in the *C*-terminal redox sites, molecular docking exhibited that a hydrogen bond formed between the LH and Met^74^, which conduced to the stable binding interaction. Consequently, we reasoned that the stronger suppression influence LH showed on Trx than on Grx might attribute to the spatially close to the redox site of Trx1. Similarly, the hydrogen bonds which LH formed with Asn^107^ and Ile^413^ of TrxR also contributed to the stability of LH-TrxR conjugated interaction.

But currently, we could not eliminate the possibility that other thiol-containing enzymes or proteins which LH would combine with. However, at least, the Trx system is one of the important targets for LH.

**Figure 5:**
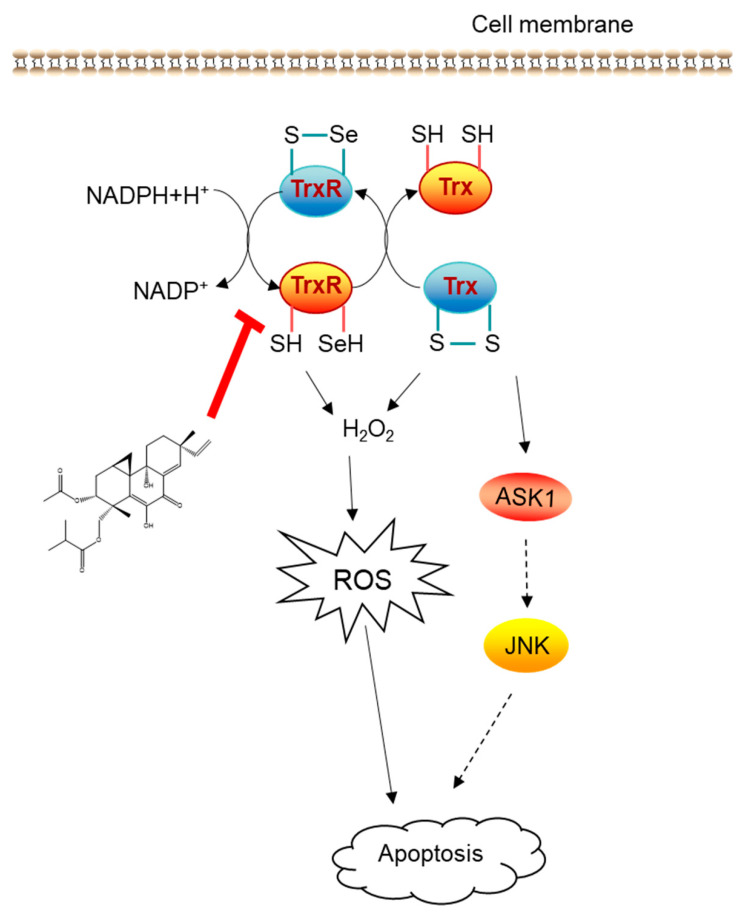
LH-mediated intracellular signaling pathway induced apoptosis in human pancreatic cancer cells.

In general, the study showed that LH was a potential antitumor compound in vitro, and was effective to against cell proliferation and pro-apoptosis in human pancreatic cancer cell lines PANC-1 and SW1990 (Figure 5). LH inhibited Trx/TrxR by a Michael addition reaction between α, β-unsaturated carbonyl moiety of LH and cysteine or selenocysteine of Trx/TrxR. The formation of the covalent bond attenuated reversible thiol/selenol reduction of Trx/TrxR, decreased Trx/TrxR activity and facilitated ROS accumulation, resulting in the dissociation of ASK1 from complex with Trx. Finally, dissociated ASK1 phosphorylation and activating downstream ASK1-JNK signaling resulted in cellular redox homeostasis disruption, and ROS-mediated cell oxidative stress, ensuring apoptosis. Our study provided important information for the explanation of the anticancer activity of pimarane diterpene and for the development of new pimarane diterpene targeting Trx system with high specificity and activity against cancer cells.

## 4. Materials and Methods

### 4.1. Reagents

LH, isolated from the Arctic fungus *Eutypella* sp. D-1 as described previously [31], was dissolved in dimethylsulphoxide as a 10 mmol/L stock solution, and diluted with culture medium. Primary antibodies anti-Trx1, anti-TrxR, anti-β-tublin, anti-JNK, anti-ASK1 were purchased from Proteintech (Wuhan, China); and anti-GAPDH, Secondary goat anti-rabbit anti-mouse IgG antibodies were from TransGen Biotech (Beijing, China). The chemoluminescence reagent was obtained from Millipore (Millipore, MA, USA). Then DCFH-DA, NAC, GSH, and SOD were purchased from Beyotime Biotechnology (Shanghai, China). All other chemical reagents were of analytical grade.

### 4.2. Cell Lines and Culture

Human pancreatic cancer cell lines PANC-1, SW1990, AsPC-1, BxPC-3 were purchased from the Cell Bank of Shanghai Institute of Biochemistry and Cell Biology. HPDE6-C7 cells was obtained from the American Type Culture Collection. Both SW1990 and AsPC-1, BxPC-3, HPDE6-C7 cell lines were maintained in RPMI 1640 medium and PANC-1 was cultured in DMEM. The cell medium was supplemented with 10% fetal bovine serum and 1% penicillin/streptomycin at 37 °C in a humidified 5% CO_2_ atmosphere incubator.

### 4.3. Cell Proliferation

Cell growth inhibition was measured by Cell Counting kit-8 assay (Solarbio, Beijing, China). Exponentially grown cells were seeded into 96-well plates at 4 × 10^3^ cells per well for overnight. Then cells were treated with a range of LH concentrations (0, 0.625, 1.25, 2.5, 10, 20, 40 µM) for 48 h. Cell viability was assessed by the absorbance at 450 nm after 10 µL CCK8 added each well and incubating at 37 °C for 45 min.

### 4.4. Cell Apoptosis Analysis

Cells were stained with Hoechst 33,258 and examined by fluorescence microscopy to examining the condensation and fragmentation of nuclei. Apoptosis ratio was quantified using flow cytometry after staining by Annexin-V-FITC/PI (KeyGEN, Nanjing, China).

### 4.5. ROS Determination

The intracellular level of ROS was examined by the oxidation sensitive fluorescent probe DCFH-DA. Exponentially cells were seeded into 6-well plate with 2 × 10^5^ cells per well for about 24 h before treated with a range of LH concentrations. Cells were harvested, stained with DCFH-DA for 30 min at 37 °C in the dark, and then measured by flow cytometer according to the manufacturer’s instructions.

### 4.6. Assessment of GSH Levels

Intracellular GSH levels were examined by using a commercial GSH and GSSG Assay Kit (Beyotime, Shanghai, China). Exponentially SW1990 cells were seeded in 6-well plate for 24 h. Different concentration of LH were added to the culture plates and continuously for indicated time. The cells were collected and deproteinized before frozen and thawed twice times with liquid nitrogen. Intracellular GSH levels were calculated on the basis of protein concentration detected by Bovine Serum Albumin (BSA) protein assay kit.

### 4.7. Western Blot Analysis

Cells were split on ice by RIPA buffer (Beyotime, Shanghai, China) containing phosphatase inhibitor (Roche, Basel, Switzerland), protease inhibitor and phenylmethanesulfonyl fluoride to collect total protein. The protein concentration was measured by BCA method. Equal amounts of the protein samples were separated by sodium dodecyl sulfate polyacrylamide gel electrophoresis (SDS-PAGE), and transferred to nitrocellulose membranes. The members were incubated with the corresponding primary and secondary antibodies after blocked in 5% nonfat milk at room temperature for 1 h. Protein expression was visualized using the Western Bright Chemiluminescent detection system.

### 4.8. Trx1 Activity Analysis

The enzyme activity of Trx1 was assessed by modified Thioredoxin Activity Fluorescent Assay Kit (Caymen Chemical, Ann Arbor, MI, USA) in 96-well plate. Various concentration of LH was added to wells containing 0.04 µM hTrx1, 1 µM TrxR and followed by addition of β-NADPH and incubated at 37 °C for 30 min. Finally, the fluorescent substrate was added, and the Trx1 activity was recorded as the emission at 545 nm after 520 nm excitation for 60 min according to the manufacturer’s instructions.

### 4.9. TrxR Enzyme Assay (Fluorescence Substrates Method)

The activity of TrxR in Trx system was performed by modified Thioredoxin Reductase Activity Fluorescent Assay Kit (Caymen Chemical, Ann Arbor, MI, USA) which based on Trx1 reduction of fluorescence substrates in cell-free system. According to the manufacturer’s instructions, the samples containing 0.4 nM TrxR and 2 µM Trx were added with 10 µL of LH at various concentrations and 5 µL of β-NADPH, followed by incubation at 37 °C for 30 min. After the addition of fluorescent substrate, recorded the emission at 545 nm after 520 nm excitation for 30 min in a fluorescent plate reader at room temperature.

### 4.10. TrxR Enzyme Assay (DTNB Method)

Method A (pre-incubation with LH): The activity of TrxR towards the reduction of DTNB was conducted in 96-well plates containing 50 mM Tris-HCL, pH 7.5, 1 mM EDTA, 20 nM TrxR, 240 µM NADPH, and 3 mM DTNB. First, 20 nM TrxR and 200 µM NADPH was incubation with varying concentrations of LH at 37 °C for 30 min. The absorbance at 410 nm was recording every 5 min for 60 min after 3 mM DTNB added.

Method B (no pre-incubation with LH): The analysis was conducted as mentioned above (Method A) except that 10 µM LH and DTNB solutions were pre-mixed and added simultaneously after reduction of TrxR by NADPH.

### 4.11. Grx1 Activity Analysis

The Grx activity assay mixture was made up of 50 μL of assay buffer (83 mM potassium phosphate, pH 7.5, containing 0.83 mM ethylene diamine tetraacetic acid (EDTA), 10 μL of NADPH, 5 μL of GSH (0.5 mM), 5 μL of Grx (the final concentration of 0.6 nM), 10 μL of GR (the final concentration of 50 nM) and 10 μL of LH at various concentrations (diluted with assay buffer). The reaction was started by the addition of 10 μL fluorescent substrate, followed by carefully shaking and monitored the emission at 545 nm after excitation at 520 nm for 30 min.

### 4.12. GR Activity Analysis

Cell-free GR activity was analyzed in 100 mM potassium phosphate (pH 7.0), 10 mM EDTA. The 100 µL reaction mixture comprised 20 µM GR, 0.75 mM DTNB, 0.1 mM NADPH, and a range of concentrations of LH. The reactions were initiated by adding oxidized 1 mM GSH and were recording at 405 nm. Activity was calculated as the increase in absorbance between 2 min after GSH addition.

### 4.13. RNA Interference

Lipofectamine 2000 (Invitrogen, Waltham, MA, USA) were used in the transference of siRNA duplexes into SW1990 cells. The siRNA sequences for TrxR were SiTrxR1-a 5′-GCAUCAAGCAGCUUUGUUATT-3′; SiTrxR1-b 5′-GGGUCCAAAUGCUGGAGAATT-3′. The siRNA sequences for GAPDH (positive control, CTL) was SiGAPDH# 5′-UGACCUCAACUACAUGGUUTT-3′. The invalid siRNA (negative control, NC) sequence was 5′-UUCUCCGAACGUGUCACGUTT-3′. Western blotting was used to assess the knockdown efficiency. Cell viability was measured by CCK8 assay as described above.

### 4.14. Molecular Docking

Molecular docking was performed using the Schrodinger software (Schrödinger, Inc., New York, NY, USA). The 3D structure of Trx1 and TrxR were retrieved from the protein data bank (PDB ID: 4PUF_C) and (PDB ID: 2zzb). In LH-TrxR1 covalent docking, the protein TrxR was prepared by Protein Preparation Tool in Schrodinger including optimized hydrogen bond network at pH 7.0 with PROKA tool, restrained minimization in OPLS3 force field with converge heavy atoms to 0.3 Å. The energy optimization of ligand LH was prepared by Ligand Preparation Tool with OPLS3 force field to produce low energy conformation. Covalent docking was performed around the reactive residue Cys497 and Cys498 within 25 Å. In LH-Trx1 covalent docking, the preparation of the protein Trx1 and LH was performed as described above except the restrained minimization was in OPLS-2005 force field before covalent docking was performed around the reactive residue Cys32 and Cys35 within 30 Å.

### 4.15. BIAM Labeling

First, TrxR was incubated with LH and 200 µM NADPH at 37 °C for 1 h. The same amounts of DMSO were treated in controls. Next, the mixture was subjected to 100 µM BIAM in 100 mM Tris–HCl and 1 mM EDTA at pH 6.5/8.5 and incubation at 37 °C for 30 min. The BIAM-labeled enzymes were denatured in nonreduced protein sample buffer and then separated by SDS–PAGE, transferred to PVDF membrane, and analysis with HRP-conjugated streptavidin and ECL.

### 4.16. MS/MS Analysis

First, 5 mM LH was incubated with 35 µg Human Trx1 protein (Sino Biological Inc., Beijing, China), and 3.3 mM LH was incubated with 4.8 µg Human TrxR protein (Cayman Chemical, Ann Arbor, MI, USA) for 2 h at 37 °C. All the protein samples were reduced by Shimadzu Biotech Proteome Kit, trypsin digested using MonoSpin Trypsin, and desalted by a MonoSpin C18 column, then detected by MALDI 7090 (SHIMADZU, Kyoto, Japan).

### 4.17. Datal Analysis

Three independent experiments were performed in triplicate. Data were analyzed by GraphPad Prism. Statistical significance was analyzed by two-tailed *t* test.

## Data Availability

The data presented in this study are available on request from the corresponding author.

## Sample Availability

Samples of the compounds are available from the authors.
